# From EXAFS of reference compounds to U(VI) speciation in contaminated environments

**DOI:** 10.1107/S1600577521013473

**Published:** 2022-02-07

**Authors:** Anna Krot, Irina Vlasova, Alexander Trigub, Alexey Averin, Vasily Yapaskurt, Stepan Kalmykov

**Affiliations:** a Lomonosov Moscow State University, Leninskie Gory 1-3, Moscow 119991, Russian Federation; b National Research Center ‘Kurchatov Institute’, Ploshchad Akademika Kurchatova 1, Moscow 123182, Russian Federation; c A. N. Frumkin Institute of Physical Chemistry and Electrochemistry, Russian Academy of Sciences, Leninskiy Prospekt 31, Moscow 119071, Russian Federation

**Keywords:** uranium, EXAFS, Raman spectroscopy, PXRD

## Abstract

Understanding the speciation of technogenic uranium in natural systems is crucial for estimating U migration and bioavailability and for developing remediation strategies for contaminated territories. Reference EXAFS data of model laboratory-prepared uranium compounds (‘standards’) are necessary to analyze U-contaminated samples from nuclear legacy sites. To minimize errors associated with measurements on different synchrotrons, it is important not only to compare data obtained on environmentally contaminated samples with the literature but also with ‘standards’ collected at the same beamline. Before recording the EXAFS spectra, all reference compounds were thoroughly characterized by Raman spectroscopy and powder X-ray diffraction. The U(VI) local molecular environments in the reference compounds, *i.e.* uranyl oxy­hydroxides, phosphates, carbonates and uranates, were examined using XAFS. Based on the EXAFS fitting results obtained, including the nature of the bonding, interatomic distances and coordination numbers, parameters that are typical for a particular U compound were differentiated. Using data for ‘standards’, U speciation in the sample of radioactively contaminated soil was determined to be a mixture of U oxyhydroxide and carbonate phases.

## Abstract 2

Multiple EXAFS *L*_III_-edge spectra of U(VI) model compounds were recorded at the STM beamline of KISI-Kurchatov (Moscow, Russia). The distinct parameters obtained for the U(VI) local structure were used to analyze the sample of radioactively contaminated soil.

## 1. Introduction

Uranium speciation in contaminated soils, groundwater, vadose zones and bottom sediments of waste storage pools governs the migration behavior in plumes of mine tailings and legacy sites of nuclear weapon production (Zachara *et al.*, 2013 ▸; Peterson *et al.*, 2018 ▸; Kaplan *et al.*, 2020 ▸; Stetten *et al.*, 2020 ▸). Decommissioning of closed enterprises and decontamination of territories require knowledge of U species to predict U migration in the environment (Maher *et al.*, 2013 ▸; Mehta, 2017 ▸; Katsenovich *et al.*, 2018 ▸). The complexity of U behavior in the environment due to the presence of carbonates, organic matter, Fe oxides, bacterial activity *etc*. requires a detailed study of individual U species under controlled laboratory conditions. In environmental systems, technogenic U is usually present as a mixture of various UO_2_^2+^ compounds or as UO_2_ particles (Křepelová *et al.*, 2008 ▸; Qafoku & Icenhower, 2018 ▸; Marshall *et al.*, 2015 ▸; Romanchuk *et al.*, 2021 ▸). Along with surface complexation with minerals and incorporation into solids like calcite, an essential mechanism of U immobilization is the formation of intrinsic U(VI) phases. The other type of UO_2_^2+^ formation in the environment is the oxidation of U(IV) phases.

Various methods can be applied for a detailed investigation of U compounds. For the characterization of environmental samples, these methods have to be nondestructive and sensitive to trace concentrations of radionuclides. The most favorable element-sensitive direct method to determine the U valence state and local atomic environment is X-ray absorption fine-structure spectroscopy (XAFS). Raman scattering µ-spectroscopy provides useful information about the U-containing phases (Lu *et al.*, 2018 ▸), although its application to natural samples is complicated due to the complex phase composition and the relatively high detection limit of the method. In the context of determining the speciation of elements in natural environments, XAFS spectroscopy is a technique with the lowest detection limits and, at the same time, the highest selectivity towards the element of interest. Additionally, Raman scattering, with no elemental selectivity, has the advantage of microscopic resolution, which makes analysis of individual micro-objects possible.

XAFS is sensitive to oxidation state (XANES, X-ray absorption near-edge structure) and local environment of U (EXAFS, extended X-ray absorption fine structure): inter­atomic distances, coordination numbers (CNs) and disorder parameter σ^2^ (Debeye–Waller factor). This method is widely used to investigate U speciation in model systems (Thompson *et al.*, 1997 ▸; Kelly *et al.*, 2007 ▸, 2010 ▸, and references therein), as well as in environmental samples such as those from Hanford liquid waste disposal sediments (Bostick *et al.*, 2002 ▸; Catalano *et al.*, 2004 ▸; Arai *et al.*, 2007 ▸), and coal and coal-combustion products in Kentucky power plants (Hower *et al.*, 2016 ▸). Many of the published spectra resolve local parameters of only axial and equatorial O (O_ax_ and O_eq_) coordination spheres. This restriction significantly limits the amount of information about U speciation that can be obtained from environmental samples.

This paper is devoted to EXAFS and µ-Raman spectra analyses of laboratory-prepared U(VI) reference compounds and, relying on obtained ‘standard’ data, analysis of U speciation in contaminated environments: a soil sample from a uranium conversion plant (Angarsk, Russia). Phosphates, carbonates and oxyhydroxides of U(VI) along with UO_2_ are solid intrinsic phases of uranium in contaminated zones of nuclear legacy sites: contaminated soils, groundwater and near-surface disposals of liquid waste (Romanchuk *et al.*, 2021 ▸). Uranates are thermodynamically stable phases under the conditions of surface reservoirs of liquid nuclear waste with high pH values.

We discuss the possibility of extending the fitting range of the U local environment to 5 Å.

## 2. Materials and methods

### 2.1. Materials

Uranyl orthophosphate tetrahydrate (UO_2_)_3_(PO_4_)_2_(H_2_O)_4_ was synthesized by the method described by Yagoubi *et al.* (2013 ▸). In the first step, 0.752 g of UO_2_(NO_3_)_2_·6H_2_O (1.5 mmol) was dissolved in 7 ml H_2_O, and then 430 µl of H_3_PO_4_ (645 mmol) was added. The mixture was kept under constant stirring for 6 h at 70°C. The solution over the precipitated pale-yellow crystals was removed with a pipette, and the remaining suspension was dried at 60°C until complete evaporation of the liquid was achieved. The crystals were sequentially washed with distilled water and acetone and dried in air.

Uranyl-ammonium carbonate (NH_4_)_4_UO_2_(CO_3_)_3_ was obtained by the following procedure (Zhang *et al.*, 2016 ▸): 0.501 g of UO_2_(NO_3_)_2_·6H_2_O (1 mmol) was dissolved in 3 ml Milli-Q H_2_O, and a yellow precipitate was formed during slow dropwise addition of 5 ml of solution containing 1.646 g (NH_4_)_2_CO_3_ (17 mmol) in Milli-Q H_2_O. The obtained supernatant was centrifuged at 7000 rpm for 2 min. The resulting yellow precipitate was decanted and dried in air.

A mixture of natural autunite Ca(UO_2_)_2_(PO_4_)_2_ and torbernite Cu(UO_2_)_2_(PO_4_)_2_ minerals was obtained from the Taboshar ore deposit, Tadjikistan.

Metaschoepite was synthesized by dissolution of 0.57 g (2 mmol) freshly prepared amorphous UO_3_ in 20 ml distilled water and stirring of the mixture at 40°C for 2 weeks. Amorphous UO_3_ was obtained from uranium peroxide UO_4_·2H_2_O. A solution of 0.01 mol UO_2_(NO_3_)_2_ was heated to 80°C, and 15 ml of 10% H_2_O_2_ was added dropwise with constant stirring. The reaction mixture was kept at 90°C for 1 h. The solution was cooled and centrifuged four times (6000 rpm, 3 min): once for separation of the precipitated peroxide and three times with water for washing. After drying at 50°C, the precipitate was placed in an oven and calcined in air at 425°C for 2 h to obtain a brown powder of amorphous UO_3_ (Watt *et al.*, 1950 ▸; Cordfunke, 1961 ▸, 1962 ▸; Cordfunke & Giessen, 1963 ▸).

Calcium uranate CaUO_4_ and diuranate CaU_2_O_7_ were prepared by the solid-state reaction of stoichiometric amounts of CaCO_3_ and U_3_O_8_ [obtained by heating UO_2_(NO_3_)_2_·6H_2_O at 900°C in air for 5 h]. The precursors were ground into a fine powder, placed into platinum crucibles and calcined three times in air for 10–20 h at 850°C. Then, the powders were pressed into pellets and calcined in the same way three more times, each time followed by crushing, thorough remixing and re-pressing into pellets (Sali *et al.*, 2000 ▸; Prieur *et al.*, 2019 ▸).

As an example of an environmental U-contaminated soil fraction studied of a nuclear legacy site, a fraction of soil collected from the area of a uranium conversion plant (Angarsk, Russia) was studied. The grain size of the sample was less than 100 µm. The mineral composition included grains of quartz, feldspar, clay minerals and, in the accessory fraction, hematite, dolomite and chlorite. The uranium concentration in the sample varied in the range from 2 mg g^−1^ to 4 mg g^−1^. A more detailed analysis of the morphology and mineral composition of contaminated soil is given by Maryakhin *et al.* (2021 ▸). In the discussion below, the sample of uranium contaminated soil will be referred to as the UCS sample.

### 2.2. Analysis

To determine the structure of the compounds studied, powder X-ray diffraction (PXRD) was used. PXRD data were collected with an Empyrean (Panalytical) diffratometer at room temperature using Cu *K*α radiation. The diffraction patterns were recorded in the 2θ range from 5° to 60° with a step size of 0.017° (2θ) and a time per step of 150 s. The *HighScore Plus* software (Degen *et al.*, 2014 ▸) was used to process the spectra and identify the phases.

Raman spectra were collected using a Renishaw inVia Raman microscope equipped with an He–Ne diode laser with an excitation wavelength of 633 nm and a diode-pumped solid-state laser with an excitation wavelength of 405 nm and Senterra Bruker microscope with solid-state Nd:YAG 532 nm laser and Ar 785 nm laser. The size of the focused spot on the sample was approximately 3 µm, and the power density was low enough to avoid surface oxidation/degradation of the samples due to local heating by the laser beam.

To determine the composition of natural minerals and obtain SEM images of the UCS sample, a JEOL JSM-6480LV scanning electron microscope (JEOL, Japan) equipped with an X-Max-50 energy-dispersive spectrometer (Oxford Instruments, UK) was used. An accelerating voltage of 20 kV and a current of 0.7 nA were used. Quantification was performed using a set of standards, listed in Table S1 of the supporting information.

X-ray absorption spectroscopy (XAS) data were recorded at the Structural Material Science (STM) beamline of the Kurchatov specialized synchrotron radiation source, KISI-Kurchatov (Moscow, Russia). Uranium *L*_III_-edge spectra were collected at room temperature in transmission mode for standards and in fluorescence mode for the UCS sample. Monochromatization of the X-ray beam was performed using a channel-cut Si (220) monochromator. To obtain high-quality experimental data, at least three scans were measured for each sample. To record EXAFS regions, a step size of 0.05 Å^−1^ was used. Each XAS scan took nearly 30 min. Energy calibration was performed using a UO_2_ reference sample between the second and third ionization chambers. Each standard sample was mixed with cellulose and pressed into a thin pellet, and the required amount of U phase to obtain qualitative data was calculated using the *HEPHAESTUS* program database. The UCS sample was pressed into a thin pellet without cellulose. Calibration and processing of the spectra obtained were carried out using the *IFEFFIT* software (Ravel & Newville, 2005 ▸). Several scans were merged and calibrated via the *ATHENA* program. The UO_2_ reference was used as a calibration standard, with a maximum of the first derivative at 17170.2 eV (Bès *et al.*, 2016 ▸). Fitting was performed via the *ARTEMIS* program. The theoretical scattering amplitude functions and phase shifts were calculated with the *FEFF6* code (Rehr *et al.*, 1992 ▸). For scattering function calculations, CIFs were retrieved from the Crystallography Open Database (COD). For CaU_2_O_7_ and (UO_2_)_3_(PO_4_)_2_·4H_2_O, structures of other compounds containing the same scattering atoms were used (CaUO_4_ and UO_2_HPO_4_, respectively). Scattering functions for the UCS sample analysis were calculated using crystallography data of schoepite, CaMgUO_2_(CO_3_)_3_ and Ca(H_3_O)_2_(UO_2_)_2_(SiO_4_)_2_. The amplitude reduction factor (*S*_0_^2^) was set to 0.9, as previously determined for the U *L*_III_-edge spectra. The shift in the threshold energy (Δ*E*_0_) was the same for all coordination spheres and varied as a global parameter. The CNs were constrained at crystallographic values where possible. In all spectra, multiple scattering paths of O_ax_—U—O_ax_ were included in the fit, with *R* and the Debye–Waller parameter defined as *R* = 2*R*(U—O_ax_) and σ^2^ = 2σ^2^(U—O_ax_), respectively. For cases of carbonates and phosphates, multiple scattering paths involving C/P and O_distant_ atoms were also included.

## 3. Results and discussion

### 3.1. PXRD data of reference samples and natural minerals mixture

To correctly interpret the EXAFS and Raman data, all studied compounds had to be free of impurities. Phase identification was carried out by PXRD. The diffractograms obtained are shown in Fig. S1 of the supporting information. Uranyl-ammonium carbonate (NH_4_)_4_UO_2_(CO_3_)_3_ and uranyl orthophosphate tetrahydrate (UO_2_)_3_(PO_4_)_2_·4H_2_O were found to be pure single-phase compounds, and the patterns were in good agreement with published data (PDF 01–073–0040 and PDF 00–037–0369, respectively). The mineral system contained three phases: metaautunite Ca(UO_2_)_2_(PO_4_)_2_·3H_2_O (00–039–1351), torbernite Cu(UO_2_)_2_(PO_4_)_2_·8H_2_O (00–036–0406) and metazeunerite Cu(UO_2_)_2_(AsO_4_)_2_·8H_2_O (01–077–0124). The presence of arsenate impurities was confirmed by scanning electron microscopy with energy-dispersive X-ray (SEM-EDX) analysis and will be discussed in more detail below. The synthesized metaschoepite contained several dehydrated phases: UO_3_·H_2_O (00–013–0242), UO_3_·0.8H_2_O (00–010–0309) and metaschoepite phase UO_3_·2H_2_O (01–070–4765). The main phase was found to be metaschoepite, and the two former phases were the products of metaschoepite reaction in air. In the discussion below, we present this synthetic sample metaschoepite [(UO_2_)_8_O_2_(OH)_12_](H_2_O)_10_, considering the presence of two dehydrated phases. Calcium uranate CaUO_4_ (01–085–0577) was found to contain diuranate impurities (00–044–0581), confirmed by Raman spectroscopy. Diuranate CaU_2_O_7_ also contains some uranate impurities of different compositions (00–022–0817).

### 3.2. SEM-EDX analysis of a natural mixture of phosphate minerals

The assumed composition of the mineral sample is a mixture of (meta-)autunite Ca(UO_2_)_2_(PO_4_)_2_·*n*H_2_O, (meta-)­torbernite Cu(UO_2_)_2_(PO_4_)_2_·*m*H_2_O and (meta-)zeunerite Cu(UO_2_)_2_(AsO_4_)_2_·*k*H_2_O. Individual crystals of Ca and Cu minerals were distinctly different in color, appearing yellow and green, respectively, as seen in the optical microscopy images (Fig. S2). The composition was clarified by SEM-EDX analysis, and the results are shown in Table S2. In autunite, a Sr impurity was observed, which was present at approximately 6 at.% Ca content, and an As impurity was present at 6–7 at.% P content. Substitutions of Ca by Sr and P by As are common in natural systems due to the similar chemical properties and effective ionic radii (Shannon & Prewitt, 1969 ▸): 1.00 Å and 1.16 Å for Ca^2+^ and Sr^2+^, and 0.17 Å and 0.34 Å for P^+5^ and As^+5^. The molar ratios of U to Ca (in areas where some Ca ions were replaced by Sr, the ratio of U to the total content of divalent metals was estimated) and to P (with a similar estimation as needed) agree well with the composition of autunite. The arsenic impurity in the torbernite part was more significant (approximately 30 at.%); however, the molar ratio of U to the sum of P and As remained consistent with the composition of torbernite. Since there were many As atoms, it was assumed that As did not isomorphically replace P in torbernite but formed its intrinsic phase.

### 3.3. Raman spectroscopy of reference samples and natural minerals mixture

Raman spectra were recorded using lasers with excitation wavelengths of 405 nm, 532 nm, 633 nm and 785 nm. Spectra recorded at optimum measurement conditions (*i.e.* the best signal-to-noise ratio and no contribution of uranium luminescence) will be discussed (633 nm or 785 nm). Band positions and their comparison with literature data are given in the supporting information (Tables S3–S7).

**Figure 1:**
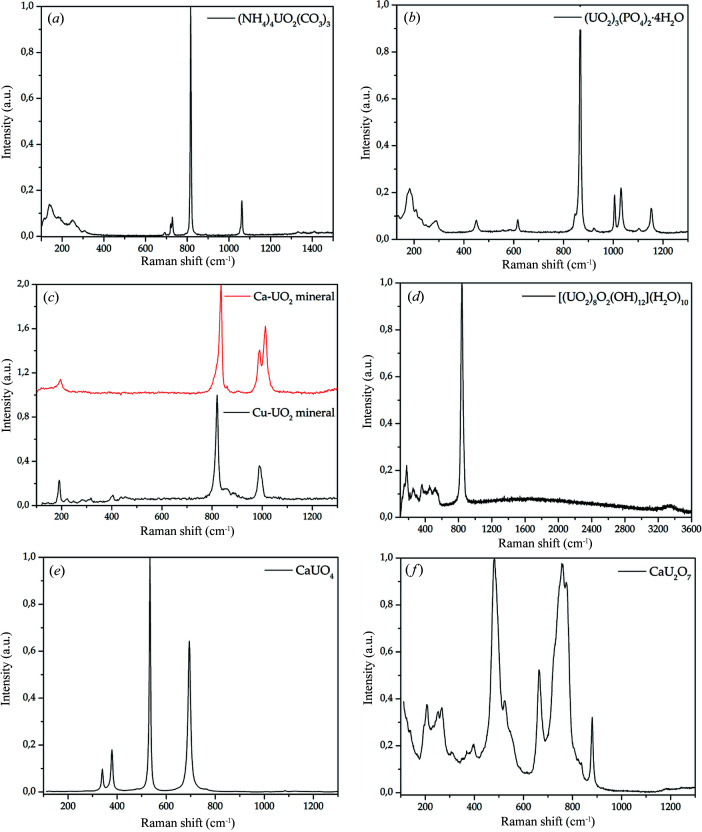
Raman spectra of (*a*) (NH_4_)_4_UO_2_(CO_3_)_3_, (*b*) (UO_2_)_3_(PO_4_)_2_·4H_2_O, (*c*) natural uranyl minerals, (*d*) metaschoepite, (*e*) CaUO_4_ and (*f*) CaU_2_O_7_.

The Raman spectrum of uranyl-ammonium carbonate is shown in Figs. 1 ▸(*a*) and S4. Vibrational data for this compound have not been published before. This spectrum can be divided into five regions: 140–300 cm^−1^, 650–900 cm^−1^, 1000–1100 cm^−1^, 1300–1600 cm^−1^ and 3450–3600 cm^−1^. The first region includes bands attributed to UO_2_^2+^ ν2 bending vibrations. Bands in the low-frequency region of the spectrum can also be attributed to vibrations of U—O_eq_ bonds and vibrations of atoms in the crystal lattice without detailed separation. The second region includes bands attributed to ν4 bending vibrations of carbonate groups at 693 cm^−1^, 721 cm^−1^ and 729 cm^−1^. The most intense band appears at 817 cm^−1^ and corresponds to uranyl ν1 symmetric stretching vibrations. Carbonate symmetric stretching vibrations correspond to a shift of the band at 1062 cm^−1^. The band is not split, which indicates the equivalence of all carbonate groups in the (NH_4_)_4_UO_2_(CO_3_)_3_ structure. Weak bands at 1334 cm^−1^ and 1408 cm^−1^ were attributed to ν3 antisymmetric stretching vibrations of carbonate groups.

Bartlett & Cooney (1989 ▸) derived an empirical equation that allows us to estimate the U=O_ax_ bond length in the UO_2_^2+^ cation from the Raman shift of the ν1 stretching vibration:

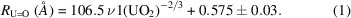

According to this equation, for uranyl-ammonium carbonate, *R*(U=O_ax_) = 1.79 ± 0.03 Å.

The Raman spectrum of uranyl orthophosphate is shown in Figs. 1 ▸(*b*) and S5. The Raman shifts are in good agreement with previously reported data (Armstrong, 2009 ▸; Pham-Thi & Colomban, 1985 ▸). The band observed at 3505 cm^−1^ corresponds to OH^−^ vibrations in H_2_O molecules. In the 900–1200 cm^−1^ region, bands of medium intensity were observed at 1005 cm^−1^, 1031 cm^−1^, 1103 cm^−1^ and 1153 cm^−1^, corresponding to PO_4_^3−^ antisymmetric stretching vibrations. A weak band at 923 cm^−1^ could be attributed to the ν1 PO_4_^3−^ vibrations or to a distortion of linearity in the uranyl cation and correspond to UO_2_^2+^ antisymmetric stretching vibrations. The strong band at 867 cm^−1^ and a nearby weak band with a shift of 846 cm^−1^ correspond to UO_2_^2+^ symmetric stretching vibrations. In the 400–700 cm^−1^ region, bands were observed at 450 cm^−1^ and 617 cm^−1^ and were attributed to ν2 and ν4 bending vibrations in the phosphate anion, respectively. Uranyl bending vibrations and vibrations of U—O_eq_ bonds gave rise to groups of bands from 136 cm^−1^ to 289 cm^−1^ in the lowest-frequency region of the spectrum.

According to equation (1), the bond length in UO_2_^2+^ is 1.75 ± 0.03 Å.

Optical microscopy imaging allowed differentiation of the Cu and Ca components of the mixture by color: green crystals corresponded to Cu minerals and yellow crystals corresponded to Ca minerals. Spectra of the two parts of the mixture were collected individually.

In the spectrum of the Cu mineral part, bands related to vibrations of both phosphate and arsenate ions were observed (Fig. S6). In the low-frequency region, bands at 191 cm^−1^, 221 cm^−1^, 247 cm^−1^ and 282 cm^−1^ were attributed to UO_2_^2+^ ν2 bending vibrations. In the 300–500 cm^−1^ region, bands were observed at 317 cm^−1^, 405 cm^−1^, 439 cm^−1^ and 455 cm^−1^. The band at 317 cm^−1^ was associated with AsO_4_^3−^ ν2 bending vibrations, at 405 cm^−1^ and 439 cm^−1^ with PO_4_^3−^ ν2 bending vibrations, and the band at 455 cm^−1^ may correspond to both ν2 vibrations of phosphate and ν4 vibrations of arsenate. The strongest band with a wide shoulder was observed at 855 cm^−1^ and corresponds to uranyl symmetric stretching vibrations. The band at 890 cm^−1^ was attributed to AsO_4_^3−^ antisymmetric stretching vibrations. The strong band at approximately 1000 cm^−1^ corresponds to the same vibrations of PO_4_^3−^.

Unlike the UO_2_–Cu pair, no arsenate minerals isostructural with phosphate were observed in the UO_2_–Ca system. In addition, according to the SEM-EDX results, the As impurity in the yellow crystals was insignificant, excluding the possibility of the formation of an intrinsic phase of uranyl-calcium arsenate. Meta-autunites of various deposits are known to contain up to 10% of the Sr substitution phase [according to the RRUFF database (http://rruff.info/)]. Therefore, taking into account the SEM-EDX and PXRD data, the yellow crystals are considered to be Ca-UO_2_ phosphate mineral autunite Ca(UO_2_)_2_(PO_4_)_2_·*n*H_2_O with minor Sr impurity.

The Raman spectrum of the autunite crystal is shown in Fig. 1 ▸(*c*). The low-frequency region contains two bands at 195 cm^−1^ and 202 cm^−1^, which are attributed to uranyl cation ν2 bending vibrations. Weak bands typical of phosphate bending vibrations in the 400–700 cm^−1^ region were not observed. The strongest broad band at ∼830 cm^−1^ was fitted (PsdVoigt1 function) by three bands at 796 cm^−1^, 818 cm^−1^ and 836 cm^−1^. These bands were attributed to UO_2_^2+^ ν1 stretching vibrations. The weak band at 860 cm^−1^ was attributed to UO_2_^2+^ ν3 antisymmetric stretching vibrations. The doublet broad band at approximately 1000 cm^−1^ was fitted using the same function by three bands at 989 cm^−1^, 1013 cm^−1^ and 1027 cm^−1^. These bands were attributed to phosphate antisymmetric stretching vibrations.

The Raman spectra confirmed the SEM-EDX data: the arsenate bands were observed and distinguished from the phosphate bands. The influence of interlayer cations on uranyl results in a 16 cm^−1^ shift of the most intense UO_2_^2+^ ν1 stretching band. In the Ca-uranyl phosphate mineral autunite spectrum, this band appears at 836 cm^−1^, and in the Cu-uranyl phosphate torbernite spectrum it appears at 820 cm^−1^. Therefore, the U—O_ax_ bond length in torbernite is expected to be longer than that in autunite. According to the Bartlett–Cooney equation (1), the distance to the O_ax_ atoms in Cu-uranyl phosphate is 1.79 ± 0.03 Å, whereas in Ca-uranyl phosphate this distance is 1.78 ± 0.03 Å. Such a difference, however, is unresolvable by EXAFS spectroscopy. The EXAFS-fitted value of 1.75 Å within the calculation and experimental errors is closer to the autunite value. This agrees with our previous findings that pointed out the predominance of the Ca-mineral phase in natural mixtures.

The spectra of both Ca and Cu parts are in good agreement with previously published data (Frost & Weier, 2004 ▸).

The spectrum of synthetic metaschoepite is presented in Fig. 1 ▸(*d*). In the range up to 600 cm^−1^, a group of weak bands was observed. These bands were attributed to UO_2_^2+^ bending vibrations, U—O_eq_ bond vibrations and lattice modes (in the range <200 cm^−1^). The strong band at approximately 850 cm^−1^ was fitted by two bands at 840 cm^−1^ and 866 cm^−1^, which were attributed to UO_2_^2+^ symmetric and antisymmetric stretching vibrations, respectively. These peak positions are consistent with the 838 cm^−1^ and 855 cm^−1^ values published by Frost *et al.* (2007 ▸) (Fig. S7). In the region corresponding to OH vibrations in H_2_O molecules, one weak broad band was observed. This indicates a distortion in the positions of water molecules. More detailed interpretation of this band is difficult due to the low signal-to-noise ratio in the corresponding spectral range. The calculated U=O_ax_ bond length is 1.77 ± 0.03 Å.

With optical magnification of the CaUO_4_ powder (Fig. S8), two phases with different colors were observed: yellow and orange. The yellow phase was predominant and represents calcium monouranate CaUO_4_ [Fig. 1 ▸(*e*)]. The spectrum contains four narrow symmetric bands at 340 cm^−1^, 379 cm^−1^, 534 cm^−1^ and 695 cm^−1^. The spectrum of the orange phase contains the same four bands corresponding to CaUO_4_ (dominant compound) and a group of bands whose positions exactly coincide with those in the spectrum of calcium diuranate CaU_2_O_7_ (Fig. S8). CaUO_4_ powder contains an impurity of calcium diuranate CaU_2_O_7_, which was confirmed by both PXRD and Raman spectroscopy.

In the CaU_2_O_7_ Raman spectrum [Fig. 1 ▸(*f*)], bands were observed at 206 cm^−1^, 251 cm^−1^, 267 cm^−1^, 308 cm^−1^, 369 cm^−1^, 398 cm^−1^, 483 cm^−1^, 525 cm^−1^, 664 cm^−1^, 725 cm^−1^, 758 cm^−1^, 780 cm^−1^ and 881 cm^−1^. The bands at 664 cm^−1^, 758 cm^−1^ and 780 cm^−1^ were attributed to stretching vibrations of primary U—O bonds, and the weak band at ∼820 cm^−1^ was attributed to ν3 antisymmetric stretching vibrations. The bands at 400–600 cm^−1^ were attributed to secondary U—O bond stretching. The bands in the 300–400 cm^−1^ region correspond to U—O bending vibrations, and low-frequency region bands may correspond to lattice vibration modes (Volkovich *et al.*, 1998 ▸, 2001 ▸).

µ-Raman spectroscopic analysis of synthesized and natural U-containing compounds was an important part of their characterization. This method requires a small amount of sample (a few milligrams) and provides a considerable amount of information at the microscale. Common features of all investigated samples include (i) strong ν1 vibration bands of UO_2_^2+^ at ∼840 cm^−1^ and (ii) a group of bands attributed to bending vibrations in the lower-frequency region 200–300 cm^−1^. By comparing the Raman shifts of the remaining bands, it is possible to distinguish uranyl compounds.

The Raman data were helpful in EXAFS fitting: by splitting the bands, we can draw conclusions regarding the equivalence or nonequivalence of functional groups in the structure and determine whether to separate CNs for the same groups or not. More importantly, Raman scattering spectra provide information about bond lengths in uranyl moieties: the interatomic distances calculated using the Bartlett–Cooney equation for all compounds were in good agreement with the values determined from the EXAFS spectra.

### 3.4. XAFS data

#### 3.4.1. Development of the fitting range

When processing the results of EXAFS experiments, the question of the accuracy of the obtained CNs and distances invariably arises. It is known that the accuracy of the calculated parameters decreases with increasing *R*. Fitting the spectra was performed in the *R*-range up to 4–5 Å. The validity of considering such a wide *R*-range and the degree of accuracy of the values obtained is unclear and requires further discussion. To estimate the accuracy of the *R* and CN calculations and to assess the maximal *R*-range, where it is acceptable, the following approach was used. It assumes that uncertainties of the fitted parameters and the maximal *R*-range, where parameters of the U local environment could be assessed with acceptable accuracy, would be similar in all samples measured at the same beamline. We chose U(IV) oxide UO_2_, which has a well known refined structure, as the reference compound and fitted its EXAFS spectrum. The EXAFS spectrum and its Fourier transform are shown in Fig. S10. In the next step, deviations of the obtained *R* values from known structural data from XRD and previous EXAFS calculations (Table S8) were assessed. The spectrum of the reference UO_2_ was recorded at the same time as the discussed U compounds. Uncertainties in the measurements in this case should be the same, which means that the range of *R* values where the parameters are determined with high accuracy is the same for all recorded spectra and the reference UO_2_. The confidential fitting range for the investigated compounds was developed by assessing the errors of the UO_2_ fitting model.

In EXAFS spectra for standard compounds with known structures, CNs are usually fixed at their crystallographic values; thus, the question of errors does not arise. In this case, only the deviation of calculated interatomic distances is of interest. Therefore, to estimate the errors, a UO_2_ fitting model with fixed CNs was constructed. The results of this fit and comparison with literature structural data are shown in Table S8. The largest difference in interatomic distances was observed in the first coordination sphere: 0.01–0.04 Å. The inconsistency of the literature data probably occurred due to point defects in the anionic sublattice. For the coordination sphere of heavy atoms, such as U, the difference between the literature structural data and the EXAFS results is smaller: the U—U distance from the EXAFS fitting coincides with the value from the structural data. Differences for longer distances are approximately 0.01–0.02 Å.

Therefore, the difference between the distances obtained from structural data and those determined from the EXAFS results does not exceed 0.04 Å for values of up to 4.5 Å. For the coordination spheres of heavy atoms, the difference is smaller and has a value of approximately 0.01 Å.

#### 3.4.2. Standard U compounds

The first step of interpreting the X-ray absorption spectra of U compounds is the determination of the U valence state. The white line and the maximum of the first derivative positions in the XANES spectra (Fig. S9) confirmed that, in all standard compounds, U is present in the +6 valence state. The determination of the valence state was of particular importance for uranates since such compounds containing uranium in a mixed-valent state are known: Cs_2_U_4_O_12_ (Berghe *et al.*, 2000 ▸, 2002 ▸), other alkali uranates *M*UO_3_ (*M* = Na, K, Rb) with a perovskite-type derivative structure (Bartram & Fryxell, 1970 ▸; Chippindale *et al.*, 1989 ▸; Soldatov *et al.*, 2007 ▸) and Ba_2_U_2_O_7_ (Alpress, 1965 ▸).

**Figure 2:**
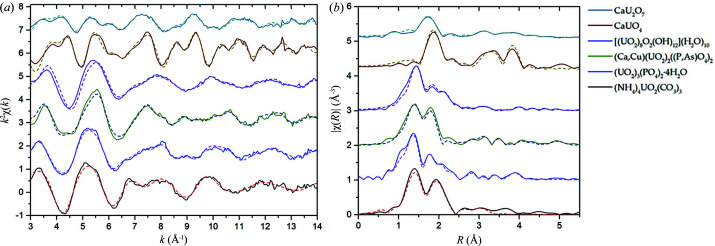
(*a*) U *L*_III_-edge *k* ^2^-weighted EXAFS spectra; (*b*) Fourier transform magnitude, not corrected for phase shifts. The dotted lines represent fit curves.

EXAFS spectra and their Fourier transforms with fitting curves are presented in Fig. 2 ▸. Fourier transforms were performed in the *k*-range from 3 Å^−1^ to 14 Å^−1^ for uranates, (NH_4_)_4_UO_2_(CO_3_)_3_ and uranyl phosphate minerals and from 3 Å^−1^ to 15 Å^−1^ for metaschoepite and (UO_2_)_3_(PO_4_)_2_(H_2_O)_4_. The calculated parameters of the local surroundings of U in standard compounds and the UCS sample are listed in Table 1 ▸. The errors provided in Table 1 ▸ were estimated in the UO_2_ fitting procedure.

The distance to O_ax_ (1.79 Å) obtained from fitting the (NH_4_)_4_UO_2_(CO_3_)_3_ EXAFS spectrum coincides with the value calculated from the Raman shift (1.79 ± 0.03 Å). In the uranyl-ammonium carbonate structure, all U positions are equivalent. Therefore, the values listed in Table 1 ▸ represent the local environment of each U atom. In the O_eq_ coordination sphere, six O atoms are equidistant because of the equivalence of the carbonate groups in the structure. The distance of 2.45 Å lies in the range typical for *M* ^+^/*M* ^2+^-UO_2_^2+^ carbonates: 2.42–2.45 Å (Catalano & Brown, 2004 ▸; Amayri *et al.*, 2005 ▸). A coordination sphere of three C atoms was added according to the structure, and the U—C distance (2.88 Å) was comparable with that of uranyl-alkali/alkali-earth metal carbonates (2.88–2.90 Å). The N coordination shell at 3.47 Å indicates the contribution of ammonium cations. The CNs for single scattering paths were constrained at the crystallographic values, and the unfitted intensity of the peaks could be attributed to the unconsidered contribution of multiple scattering paths.

In uranyl orthophosphate, the distance of U—O_ax_ obtained from the fitting of EXAFS spectra (1.75 Å) coincides with the value obtained from the analysis of the Raman spectrum (1.75 ± 0.03 Å). Fitting of all the coordination spheres of U in synthetic (UO_2_)_3_(PO_4_)_2_·4H_2_O was performed according to the known uranyl orthophosphate structure. It contains U in two nonequivalent positions: intralayer (within the layer) U and interlayer U in the ratio of 2:1. Intralayer U is present in pentagonal-bipyramid polyhedra surrounded by four phosphate tetrahedra, creating uranyl phosphate sheets. This intralayer uranyl is bound to three monodentate phosphate groups and one bidentate phosphate group. The sheets formed in this way are joined by uranyl polyhedra in the interlayer, creating a framework structure. Interlayer uranyl is bound to two phosphate anions of neighboring sheets in a monodentate manner. The EXAFS spectrum represents the average local environment of U in two positions. The environment around U in the interlayer and intralayer positions contributes to CN values with coefficients of 1/3 and 2/3, respectively (according to the 2:1 ratio of these types of U in the structure). O atoms in the equatorial plane at distances of 2.27 Å and 2.35 Å are attributed to intralayer uranium positions. The shorter interatomic distance with CN = 2 × 2/3 = 4/3 is attributed to two O atoms in the bidentate phosphate anion, and longer distances with CN = 3 × 2/3 = 2 are attributed to three O atoms in monodentate phosphate. The CNs were multiplied by 2/3 according to the structure to consider the nonequivalence of U positions (the coefficient for U in the intralayer positions mentioned above). Note that with the given *k* range (3–15 Å^−1^, Δ*k* = 12 Å^−1^) the expected resolution in *R*-space is approximately 0.13 Å. The difference in calculated distances to O_eq_ subshells in uranyl orthophosphate is <0.13 Å, which might indicate higher uncertainties in these values. The P coordination shell is split into monodentate and bidentate phosphate subshells at distances of 3.13 Å and 3.84 Å, where the bidentate phosphate is closer than the monodentate phosphate. CNs were calculated in the same way as the equatorial O coordination sphere. The P subshells at 3.13 Å and 3.84 Å are attributed to phosphates around uranyl in the intralayer position: one bidentate and three monodentate bound anions. Therefore, the CNs should be multiplied by 2/3. The P subshell at 3.64 Å with CN = 2 × 1/3 contains two monodentate phosphate anions bound to uranyl in the interlayer position. For monodentate phosphate in an autunite-type structure (layers of uranyl octahedra connected to four phosphates in a monodentate manner), common U—P distances are approximately 3.6 Å. In the case of uranyl orthophosphate, the distance to the phosphate anions in the sheet is longer. The value of 3.84 Å is in good agreement with the result of Catalano & Brown (2004 ▸) for the same orthophosphate (3.80 Å) and for phosphuranylite (3.81 Å), which also represents a framework structure (Demartin *et al.*, 1991 ▸). A more distant U subshell (4.02 Å) is attributed to two intralayer U neighbors. The uranium coordination shell is split into two subshells: one with CN 2 × 2/3 at a distance of 4.02 Å and one with the same CN at a longer distance, which is beyond the detection limit and was not fitted. The total CN for the nearest U coordination shell within the sheet is 4. In the interlayer position, the U—O_eq_ distance is 2.46 Å, greater than that for intralayer atoms. This result is reasonable, as chemical bonds within the sheet are stronger than those between the interlayer uranyl and phosphates of neighboring sheets. Water molecules were added to the model as ligands that saturate the coordination sphere of interlayer U (otherwise, the CN of interlayer U would be 2, which is chemically unrealistic). The best fit was achieved after the addition of 2 × 1/3 H_2_O, resulting in 4-coordinate U in the interlayer.

The studied natural minerals were identified as a mixture of Ca(UO_2_)_2_(PO_4_)_2_ and Cu(UO_2_)_2_(PO_4_)_2_ hydrates with a predominant Ca-phase and a Cu(UO_2_)_2_(AsO_4_)_2_ impurity phase. The minerals were isostructural; therefore, the U atoms have identical environments, differing only in the partial substitution of P for As and in interlayer cations. EXAFS spectroscopy is sensitive to the main phases and represents the average local U environment. According to SEM-EDX results (Table S2), As-substituted torbernite is about 1/3 of the total amount of Cu-minerals. At the same time, Cu phases do not exceed 20% of the mixture since the main phase is Ca-mineral autunite. Total As content is <7%. Therefore, the contribution of As impurities to the EXAFS spectrum is negligible. Interlayer cations also contribute slightly to the EXAFS data since their distribution around the absorbing atom is not regular and their scattering properties are weak. The addition of Ca and Cu to the fitting model did not improve the fit. Taking into account the negligible contribution of interlayer cations and As in the EXAFS spectrum, three species present in the natural mixture of isostructural autunite-type minerals cannot be distinguished by EXAFS spectroscopy. The spectrum of this sample was considered the standard of U in an autunite-type structure. The U—O_ax_ distance (1.75 Å) is less than the typical value for layered autunite-type minerals (1.77–1.78 Å) but coincides with the result for synthetic orthophosphate (UO_2_)_3_(PO_4_)_2_·4H_2_O. The distance to the equatorial O coordination sphere (2.27 Å) also coincides with the value calculated for monodentate coordinated phosphate in synthetic (UO_2_)_3_(PO_4_)_2_·4H_2_O and agrees well with literature data (2.28–2.29 Å). The O coordination shell at 3.49 Å reflects the contribution of interlayer water molecules. The equatorial O shell and P coordination shell in all three minerals of the natural mixture appear at approximately the same distances. The addition of distant O coordination spheres at 3.96 Å and 4.76 Å and multiple scattering paths associated with P and distant O significantly improved the model. The coordination sphere of four neighboring U atoms appears at 5.15 Å. The calculated value is close to that obtained previously by Catalano & Brown (2004 ▸) for various autunite-type minerals (∼5.2 Å).

The distance to both axial and equatorial O coordination spheres in minerals coincides with that in uranyl phosphate and is reduced in comparison with that of carbonate. At the same time, the P shell is located farther (for bidentate and monodentate phosphate, the distances are ∼3.15 and 3.6–3.8 Å, respectively) than the C shell in carbonate (∼2.9 Å). Relying on interatomic distances, it is possible to differentiate between monodentate and bidentate coordinated phosphate ions: the bidentate-bound P coordination sphere is located at a distance of ∼3.15 Å, whereas the monodentate coordination sphere is located at a distance of 3.6–3.8 Å. Monodentate phosphates connected to uranyl in layered structures are closer and appear at ∼3.6 Å, whereas in framework structures a larger distance of ∼3.8 Å to the monodentate-bound phosphates was observed. There are significant differences between autunite-type phosphates, uranyl orthophosphate and carbonates in axial and equatorial O coordination shells.

According to the XRD data, the synthesized oxyhydroxide sample consists of predominantly metaschoepite and two additional dehydrated phases. The structural similarity of these three phases within a layer allows us to interpret the interatomic distances (*R*) calculated from the EXAFS spectra as correct parameters specific for uranyl oxyhydroxide compounds and use the spectrum as the standard of U in the oxyhydroxide structure. U oxyhydroxides are layered compounds that contain U—O sheets with U atoms and pentagonal-bipyramidal coordination, joined together by interlayer water molecules. In the metaschoepite structure, compared with schoepite (Finch *et al.*, 1997 ▸), the two most weakly bound water molecules are removed from the interlayer space, and ten H_2_O molecules remain. The structure of the layers does not change on conversion from schoepite to metaschoepite, and the cell parameters change only slightly: *a* decreases by ∼0.3 Å. During the transformation of metaschoepite to dehydrated schoepite, all interlayer water molecules are removed and the layers recrystallize. The sheets of both (meta-)schoepite and dehydrated schoepite represent α-UO_2_(OH)_2_-type structures. Metaschoepite is characterized by the presence of ordered anionic vacancies at a 1:8 ratio, and the dehydrated schoepite vacancies are disordered. The compositions of the layers in metaschoepite and dehydrated schoepite are the same, and UO_3_·0.75H_2_O and U atoms adopt fivefold coordination. The structures of three oxyhydroxide phases in our sample within the layer differ only in the ordering of the O vacancies. Therefore, the EXAFS spectrum correctly describes the local surroundings of U atoms in the oxyhydroxide layers, as the ordering of vacancies is not detectable by EXAFS spectroscopy. The interlayer space differs by the number of remaining water molecules, which will affect only the CN of interlayer H_2_O in the EXAFS model. Despite the presence of several phases in our sample, it could be used as a standard of U in oxyhydroxide-type structures. The calculated parameters for the metaschoepite [(UO_2_)_8_O_2_(OH)_12_](H_2_O)_10_ sample are presented in Table 1 ▸. The distance to the axial O coordination sphere (1.80 Å) within its uncertainty agrees with the value obtained from the Raman spectrum (1.77 ± 0.03 Å). The distances to the axial and equatorial O subshells agree well with known structural data and are similar to typical values from the literature (Allen *et al.*, 1996 ▸; Froideval *et al.*, 2006 ▸; Aamrani *et al.*, 2007 ▸). The uranium coordination sphere is also split: it is fitted by four U atoms at different distances in the range 4–5 Å. The O coordination shell at 3.46 Å corresponds to interlayer water molecules. The best fit was achieved by fixing the CN for this shell to 1.

Unlike other U^+6^ compounds, uranates do not contain uranyl UO_2_^2+^ ions in their structure. That is, there are no strongly bound axial oxygen atoms at distances of ∼1.8 Å. In the structure of calcium monouranate CaUO_4_, layers and chains of UO_8_ hexagonal bipyramids alternate with Ca—O layers. For two oxygen atoms in the axial plane of U-polyhedra, a distance larger than that for axial O in uranyl is characteristic. The calculated values of 1.94 Å and 2.27 Å for axial and equatorial oxygen coordination spheres are in good agreement with the EXAFS and PXRD data obtained by Prieur *et al.* (2019 ▸). For more distant coordination spheres, the CNs and interatomic distances have not been previously published. Our calculated distance to the neighboring U coordination shell (3.88 Å) coincides with the *a* and *b* parameters of the crystal lattice, as reported by Prieur and co­authors; in the CaUO_4_ structure, *a* = *b* = 3.87745 Å (Prieur *et al.*, 2019 ▸). The U atoms in layers are located at the vertices of the rhombus. In chains, due to geometry, the U atoms are at the same distances. The CN of U was calculated with consideration of the following points: the number of neighboring atoms around U is six in the layers and four in the chains at a distance equal to *a*. The positions in the layers are occupied by 1/3 of all the atoms, and the remaining 2/3 of U atoms are in chains. Hence, the CN of U is equal to (6 × 1/3) + (4 × 2/3) = 14/3. The CN for Ca was calculated similarly: CN_Ca_ = (2 × 1/3) + (4 × 2/3) = 10/3. The CN for O atoms at 3.44 Å was treated as a variable, as it is difficult to assess correctly.

In the case of Ca diuranate, an accurate fit with fixed CNs was complicated due to the lack of data on the structure and previously performed calculations for calcium diuranate. EXAFS calculations were previously performed for diuran­ates of other metal cations: K^+^, Cs^+^ and Ba^2+^ (Table 2 ▸). However, it is not expected that CaU_2_O_7_ has a similar structure and hence a similar local U environment. Therefore, during the fitting of CaU_2_O_7_, the CNs for all coordination spheres (except the first one with two O atoms in the axial plane) were treated as variables owing to the lack of crystallographic data for this structure or previously performed EXAFS calculations. Several attempts have been made to construct a realistic fitting model; coordination spheres of O, Ca and U were added in different variations to fit peaks in the 3–5 Å region. In Table 1 ▸, the parameters of the best fitting model are listed. The distance to the axial O (1.88 Å) is greater than that in uranyl compounds (1.76–1.82 Å). A longer distance to the first coordination shell compared with that of UO_2_^2+^ compounds is typical for uranates and is the most obvious and easily detectable distinctive feature of uranates. The Debye–Waller factor of axial oxygens is greater than that for uranyl compounds: O atoms in the axial plane in uranates are less ordered and weakly bound to U. The second coordination shell is split and contains four to five O atoms in the equatorial plane at distances of 2.08 Å and 2.21 Å, which are less than those of other metal uranates (Table 2 ▸). A significant difference from K, Cs and Ba diuranates is the order of the coordination spheres: in our case, the Ca shell is located closer than the U shell. At the same time, in CaU_2_O_7_, the distances to the U shell are greater than those known for other diuranates. Notably, the calculated values could be affected by the impurity of other Ca uranates, which was observed in the XRD data. The accuracy of the obtained values is reduced due to this factor.

### 3.5. Sample of uranium contaminated soil

**Figure 3:**
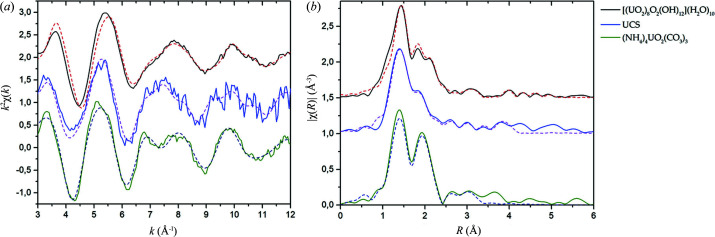
(*a*) U *L*_III_-edge *k* ^2^-weighted EXAFS spectra of the UCS sample with schoepite and carbonate standards; (*b*) Fourier transform magnitude, not corrected for phase shifts. The dotted lines represent fit curves.

The sample of uranium-contaminated soil (UCS) mainly consists of clay minerals, quartz and feldspar with a particle size <100 µm (Maryakhin *et al.*, 2021 ▸). SEM images show the presence of 3–7 µm particles and aggregates with high U concentrations (Fig. S3). SEM-EDX analysis demonstrates a positive correlation between Ca and U content. These observations propose the formation of uranyl-Ca mineral phases in the contaminated soil. XANES shows that, in the UCS sample, U is present in the +6 valence state. The shoulder observed in the post-edge region indicates the formation of the uranyl cation UO_2_^2+^ (Fig. S9). According to EXAFS fitting results, U speciation in the UCS sample is controlled mainly by the formation of uranyl oxyhydroxide and to a lesser extent by carbonate phases. Distances to O_ax_ and O_eq_ in the UCS sample are in excellent agreement with those obtained for the metaschoepite standard. The formation of metaschoepite is additionally supported by the presence of the U coordination shell at 3.97 Å. The coordination shell of C at 2.90 Å may be attributed to the complexation with natural organic matter of the soil or the formation of uranyl carbonate intrinsic phases. Although U–humate complexation through carboxyl groups cannot be completely excluded in the UCS sample, the formation of uranyl carbonates seems to be more likely, as we found that U distribution correlates with Ca and thus Ca-uranyl carbonate species such as libegite Ca_2_[(UO_2_)_2_(CO_3_)_4_] are assumed. U—O_eq_ shell splitting and U—U distance also might indicate the formation of uranyl silicates (Catalano & Brown, 2004 ▸). However, addition of Si subshells to the fitting model resulted in close to zero CNs of these subshells. Uranyl silicates are improbable phases in the UCS sample. A comparison of *k* ^2^χ(*k*) and Fourier transform spectra of the UCS sample with oxyhydroxide and carbonate is given in Fig. 3 ▸.

### 3.6. Environmental implications

The main aim of geometry refinement via EXAFS of compounds with known structures is the determination of specific parameters (interatomic distances) and their combinations typical only for a given uranyl structure. By comparing the fitted values of contaminated natural samples with standards, one can assess U speciation in investigated natural samples. This approach was implemented to determine U speciation in the UCS sample. The determination of U speciation in nuclear legacy sites is essential for controlling the migration ability, preventing contamination and remediation.

All standard compounds discussed are possible under environmental conditions. The formation of uranates was predicted by thermodynamic modeling under conditions of some of the surface disposal of liquid radioactive waste. The formation of uranates could be confirmed by EXAFS spectroscopy by observing O atoms in the axial plane at an increased distance of 1.88–1.94 Å, which is unique to uranates and not observed in uranyl compounds.

Specifically, the mixture of natural autunite-type minerals (Ca,Cu)(UO_2_)_2_[(P,As)O_4_]_2_ should be pointed out. Although the sample is a mixture of several uranyl phases, it could be used as a standard for uranyl in autunite-type structures, the most common structure among uranyl phosphate minerals.

The application of Raman spectroscopy to environmental samples is complicated. The presence of large amounts of minerals in natural samples prevents the observation of bands of minor phases, such as U contamination. However, the use of Raman spectroscopy to identify U compounds in bottom sediments of liquid waste storage, slurry storage, uranium mining tailings and other objects with a high uranium content seems to be completely justified.

The determination of U speciation is important in terms of rehabilitation and nuclear waste management. In the case of contaminated natural samples of unknown composition, extended research applying a number of analytical approaches is required to develop an accurate EXAFS fitting model and establish U speciation.

## 4. Conclusions

Several U(VI) synthesized compounds, including (NH_4_)_4_UO_2_(CO_3_)_3_, orthophosphate (UO_2_)_3_(PO_4_)_2_·4H_2_O, oxyhydroxide [(UO_2_)_8_O_2_(OH)_12_](H_2_O)_10_, uranate CaUO_4_, diuranate CaU_2_O_7_ and a natural mixture of autunite-type minerals (Ca,Cu)(UO_2_)_2_[(P,As)O_4_]_2_, were investigated by PXRD, EXAFS and Raman spectroscopy. Based on the obtained EXAFS data regarding the nature, interatomic distance and CNs of atoms in the nearest U surrounding, we identified parameters that are common for a particular U compound. All standard compounds studied have specific features that are typical only for particular structures. The first feature is the distance and splitting of the O_eq_ coordination sphere: carbonates in the [UO_2_(CO_3_)_3_]^4−^ structure do not exhibit O_eq_ sphere splitting, have CN = 6 and distances that are greater than those observed for 4-coordinate U in the autunite-type structure, which also does not exhibit O_eq_ sphere splitting. Notably, only interatomic distances are useful for comparison with environmental samples. Natural samples usually contain a mixture of several species, and their average CNs are different from the values in pure compounds. Second, the distances to central atoms in surrounding anions are characteristic. Carbonates can be easily distinguished: a distance of ∼2.9 Å is unique and typical only for the C coordination sphere. Another more complicated question is whether these ligands are inorganic carbonates or organic groups from natural organic matter. To answer this question, additional investigation of the spectrum is required. In some cases, it is possible to assess the C_org_:C_inorg_ ratio by including multiple scattering paths and O_distant_ coordination spheres with varying CNs; small CNs for these coordination shells will indicate a smaller contribution of carbonates and the pre­dominance of organic groups, and vice versa. In the example of (UO_2_)_3_(PO_4_)_2_·4H_2_O, we showed that it is possible to differentiate monodentate and bidentate coordination of phosphate: for bidentate coordination, the typical distance is ∼3.2 Å, and for monodentate coordination this distance is 3.6–3.8 Å. Third, distances to neighboring U atoms provide useful information. U subshells at ∼4–4.3 Å and ∼4.8–5 Å are typical for oxyhydroxide-type structures. An indication of an autunite-type structure (along with distances to the O_eq_ and P atoms) is the presence of a U coordination sphere at ∼5.20 Å. In comparison, in uranyl orthophosphate, a U coordination sphere appears at ∼4 Å. Moreover, no contribution of neighboring U is observed in the case of uranyl-ammonium carbonate.

By comparing characteristic features of standard U compounds mentioned above with U local surrounding parameters of the UCS sample and applying additional analytical techniques such as SEM-EDX, U speciation in the contaminated soil was determined as predominantly uranyl oxyhydroxide phase and carbonate of the uranyl-calcium phase.

The obtained EXAFS data on the standards will help us to estimate U speciation in natural contaminated samples. Additional structural information regarding U-containing phases may be provided by PXRD, SEM-EDX and µ-Raman spectroscopy in the case of high U concentrations in radioactive wastes. Based on the knowledge of U speciation in nuclear legacy sites, it will be possible to control U contamination in the environment by creating immobilization barriers, establishing appropriate disposal conditions and developing rehabilitation strategies.

## 5. Related literature

The following references, not cited in the main body of the paper, have been cited in the supporting information: Allen & Griffiths (1979 ▸); Boyanov *et al.* (2007 ▸, 2017 ▸); Denecke *et al.* (2005 ▸); Faulques *et al.* (2018 ▸); Frost *et al.* (2004 ▸); Jovani-Abril (2014 ▸); O’Loughlin *et al.* (2003 ▸); Opel *et al.* (2007 ▸); Wasserstein (1951 ▸).

## Supplementary Material

Supporting figures and tables. DOI: 10.1107/S1600577521013473/ok5063sup1.pdf

## Figures and Tables

**Table 1:** Calculated interatomic distances, coordination numbers and Debye–Waller factors Bold values were treated as fixed parameters during fitting.

		U—O_ax_			U—O_eq_						
Compound	Δ*E*_0_ (eV)	CN†	*R* ‡ (Å)	σ^2^ (Å^2^)	CN	*R* (Å)	σ^2^ (Å^2^)		CN	*R* (Å)	σ^2^ (Å^2^)
(NH_4_)_4_UO_2_(CO_3_)_3_	3.6	**2**	1.79	0.001	**6**	2.45	0.006	C	**3**	2.88	0.005
								N	**4**	3.47	0.008
(UO_2_)_3_(PO_4_)_2_(H_2_O)_4_	6.4	**2**	1.75	0.001	**4/3**	2.27	0.003	O	**2/3**	2.88	0.003
					**2**	2.35		P	**2/3**	3.13	0.003
					**4/3**	2.46			**2/3**	3.64	0.003
									**2**	3.84	0.004
								U	**4/3**	4.02	0.006
(Ca,Cu)(UO_2_)_2_(PO_4_)_2_	7.0	**2**	1.75	0.003	**4**	2.27	0.003	O	**4**	3.49	0.003
								P	**4**	3.56	0.006
								O	**8**	3.96	0.010
								O	**4**	4.76	0.006
								U	**4**	5.15	0.007
[(UO_2_)_8_O_2_(OH)_12_](H_2_O)_10_	13.4	**2**	1.80	0.002	**2**	2.32	0.004	O	**1**	3.47	0.003
					**3**	2.47		U	**1**	4.05	0.006
									**1**	4.28	
									**1**	4.75	
									**1**	4.95	
CaUO_4_	12.6	**2**	1.94	0.004	**6**	2.27	0.005	O	2.2	3.44	0.003
								Ca	**10/3**	3.66	0.004
								U	**14/3**	3.88	0.004
CaU_2_O_7_	−3.0	**2**	1.88	**0.003**	1.5	2.08	0.003	Ca	2.4	3.77	0.007
					3.3	2.21			3.3	4.16	
								U	2.6	4.22	0.005
									1.5	4.45	
UCS	8.5	**2**	1.80	0.001	3.5	2.30	0.004	C	2.3	2.90	0.003
					2.7	2.47		O	2.3	3.96	0.003
								U	2.0	3.97	0.006

† Errors in CNs are ±25%.

‡ Errors in distances do not exceed 0.04 Å.

**Table 2:** Distances to the nearest coordination spheres in Cs, Ba and K diuranates

Compound	U—O_ax_	U—O_eq_	U—O_eq2_	U—O_eq3_	U—U	U—Me	Reference
Cs_2_U_2_O_7_ (PXRD)	1.81	2.16	2.35	2.46 (×2)	3.62		Mijlhoff *et al.* (1993 ▸)
	1.84						
Cs_2_U_2_O_7_ (EXAFS)	1.94	2.17	2.38				Berghe *et al.* (2002 ▸)
BaU_2_O_7_ (PXRD)	1.84	2.12 (×2)	2.33 (×2)		3.56 (×2)	4.23 (×4)	Alpress (1965 ▸)
					3.81 (×2)		
K_2_U_2_O_7_	1.85	2.15 (×2)	2.21 (×2)		3.65	3.96 (×3)	Saine (1989 ▸)
	1.93				3.86	4.10 (×3)	
					3.94		
					4.00		
