# Supplementary material for: From EXAFS of reference compounds to U(VI) speciation in contaminated environments
Source: J Synchrotron Radiat. 2022 Feb 7;29(Pt 2):303–14. doi: 10.1107/S1600577521013473 (PMC8900840; doi:10.1107/S1600577521013473)
Supplement: Supplementary file 1 [file s-29-00303-sup1.pdf]

JOURNAL OF SYNCHROTRON RADIATION

Volume 29 (2022)

Supporting information for article:

From EXAFS of reference compounds to U(VI) speciation in contaminated environments

Anna Krot, Irina Vlasova, Alexander Trigub, Alexey Averin, Vasily Yapaskurt and Stepan Kalmykov

S1. Powder X-ray diffraction

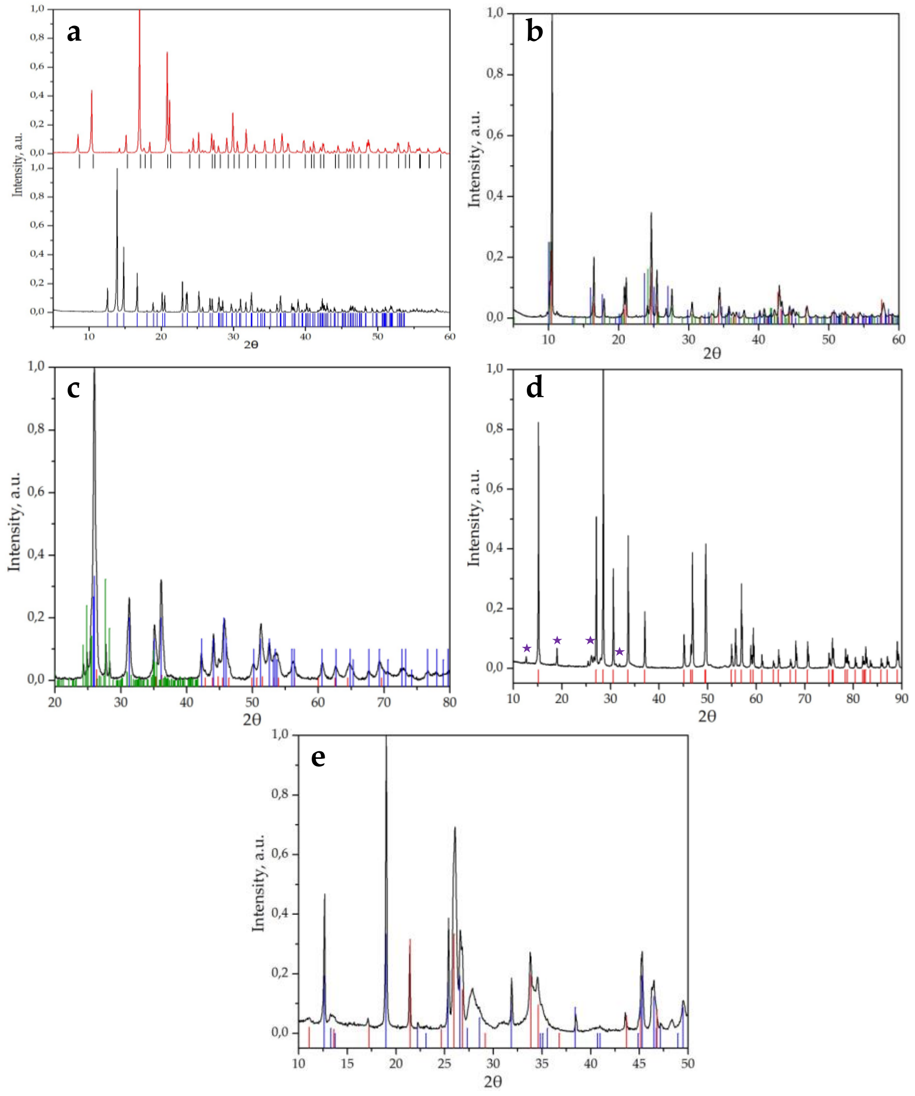

Figure S1 Powder X-ray diffraction of: (**a**) synthetized $(UO_2)_3(PO_4)_2{\cdot}4H_2O$ (red) and $(NH_4)_4UO_2(CO_3)_3$ (black). Reflections are in a good agreement with PDF 00-037-0369 and PDF 01-073-0040, respectively; (**b**) mixture of natural phosphate minerals. Red patterns corresponds to metaautunite $Ca(UO_2)_2(PO_4)_2{\cdot}3H_2O$ (PDF 00-039-1351), green to torbernite $Cu(UO_2)_2(PO_4)_2{\cdot}8H_2O$ (PDF 00-036-0406) and blue to metazeunerite $Cu(UO_2)_2(AsO_4)_2{\cdot}8H_2O$ (PDF 01-077-0124); (**c**) synthetized uranium oxyhydroxide. Three phases were identified: metaschoepite $UO_3{\cdot}2H_2O$ (PDF 01-070-4765) – green pattern, and dehydrated phases $UO_3{\cdot}H_2O$ (PDF 00-013-0242) – red pattern, $UO_3{\cdot}0.8H_2O$ (PDF 00-010-0309) – blue pattern; (**d**) $CaUO_4$. Red patterns correspond to $CaUO_4$ structure (PDF 01-085-0577), purple stars indicate reflections of impurity CaU_2O_7 phase (PDF 00-044-0581); (**e**) CaU_2O_7. Blue patterns correspond to CaU_2O_7 phase (PDF 00-044-0581), red – to impurity of uranate of different composition, $CaU_5O_{15.4}$ (PDF 00-022-0817).

S2. SEM-EDX

Table S1 Standards for EDX quantitative analysis.

**Element**	**P**	**Ca**	**As**	**Cu**	**Sr**	**U**
series	K	K	K	K	K	M
standard	GaP	Cpx-164905	InAs	Cu	SrF_2	UO_2
Sigma, wt%	0.02	0.02	0.02	0.03	0.10	0.20

Table S2 EDX data on mineral system composition, at.%. "Autunite" represents points, recorded on yellow crystals which consist of Ca-UO_2 phosphate mineral, "Torbernite" – green crystals of Cu-UO_2 mineral. Oxygen calculated by stoichiometry.

**Point**	**All results in atomic %**								
	**P**	**As**	**Ca**	**Cu**	**Sr**	**U**	**O**	**U:(P+As)**	**U:(Ca+Sr)**
Torbernite-1	7.94	3.89		5.91		11.69	70.57	1.0	2.0
Torbernite-2	7.83	4.1		5.61		11.76	70.7	1.0	2.1
Torbernite-3	8.25	3.95		5.4		11.62	70.77	1.0	2.2
Torbernite-4	10.37	1.5		5.17		12.03	70.93	1.0	2.3
Autunite-1	11.25	0.7	5.52		0.34	11.61	70.58	1.0	2.0
Autunite-2	10.73	0.83	5.68		0.33	11.88	70.55	1.0	2.0

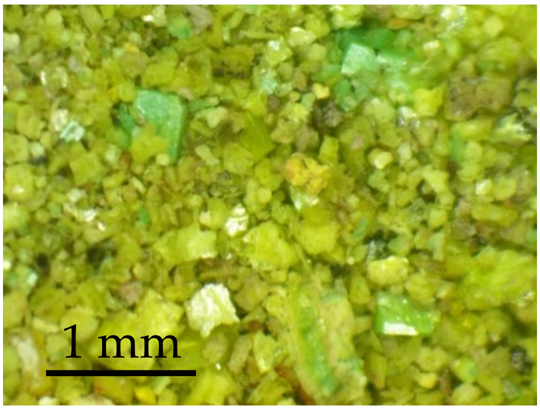

Figure S2 Optical image of the mixture of phosphate minerals. Yellow crystals represent Ca-uranyl phosphate, green – Cu-containing phases.

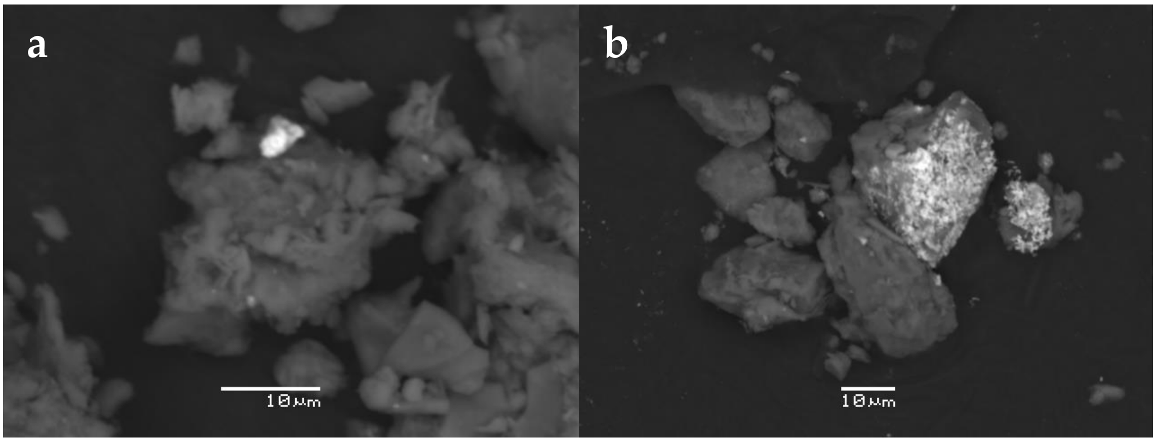

Figure S3 SEM images of particles (a) and coatings (b) with high U content in the UCS sample.

S3. Raman spectroscopy

Table S3 Raman shifts of $(NH_4)_4UO_2(CO_3)_3$ compared to literature data for similar compounds. Ionic charges are omitted for simplicity.

**Compound**	**$(NH_4)_4UO_2(CO_3)_3$**	**$Na_2Ca(UO_2)(CO_3)_3 \cdot xH_2O$**	**$Ca_2Cu(UO_2)(CO_3)_4 \cdot 6H_2O$**
ν2 (UO_2)<br>ν (U-$O_{eq.}$)<br>δ (U-$O_{eq.}$)	142, 185, 249, 276, 309	164, 182, 224, 242, 272, 284, 299	140, 169, 212, 230, 266
ν4 (CO_3)	693, 721, 729	696, 742	731, 756
ν1 (UO_2)/ν2 (CO_3)	817	831, 833	826, 834, 840
ν3 (UO_2)		(IR)	(IR 897)
ν1 (CO_3)	1062	1080, 1092	1074, 1092
ν3 (CO_3)	1334, 1408	1370, 1406	1381, 1566
H_2O		3415, 3510, 3558	3240, 3290, 3383, 3495
Reference	This work	Frost et al. (2004)	Faulques et al. (2018)

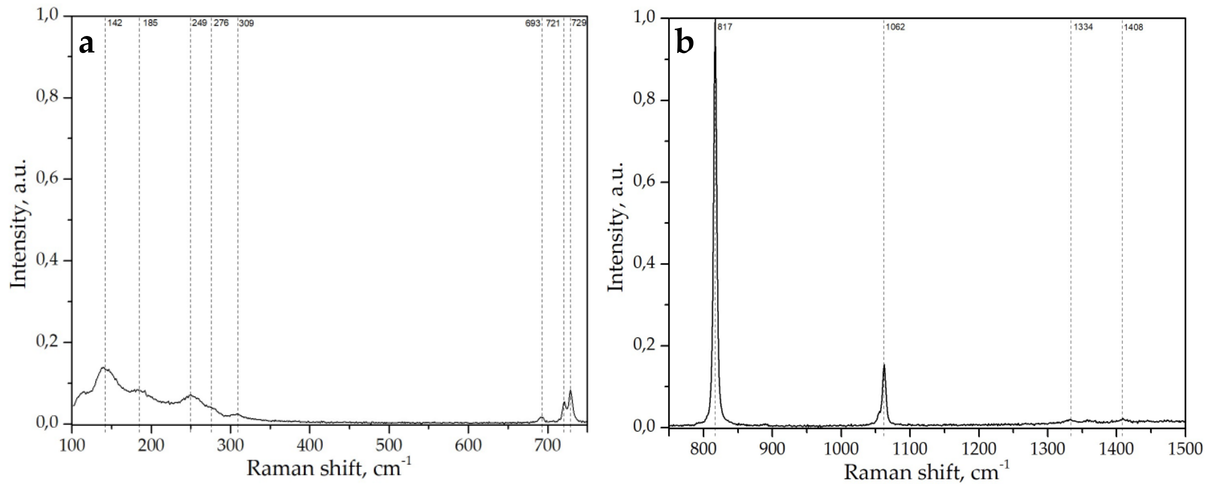

Figure S4 Raman spectrum of $(NH_4)_4UO_2(CO_3)_3$, recorded at 633 nm. (**a**) 100-750 cm^{-1} region; (**b**) 750-1500 cm^{-1} region.

Table S4 Raman shifts of $(UO_2)_3(PO_4)_2{\cdot}4H_2O$ compared to literature data for similar compounds. Ionic charges are omitted for simplicity.

**Compound**	**$(UO_2)_3(PO_4)_2{\cdot}4H_2O$**	**$(UO_2)_3(PO_4)_2{\cdot}4H_2O$**	**$(UO_2)_3(PO_4)_2{\cdot}4H_2O$**
ν2 (UO_2)<br>ν (U-$O_{eq.}$)<br>δ (U-$O_{eq.}$)	182, 208, 219, 230, 246, 289	289	185, 290
ν2 (PO_4)	450		450
ν4 (PO_4)	617		620
ν1 (UO_2)	846, 867	868	868
ν3 (UO_2)/ν1 (PO_4)	923		916
ν3 (PO4)	1005, 1031, 1103, 1153	1005, 1033, 1153	1007, 1035, 1115, 1153
H_2O	3505		
Reference	This work	Armstrong (2009)	Pham-Thi&Colomban (1985)

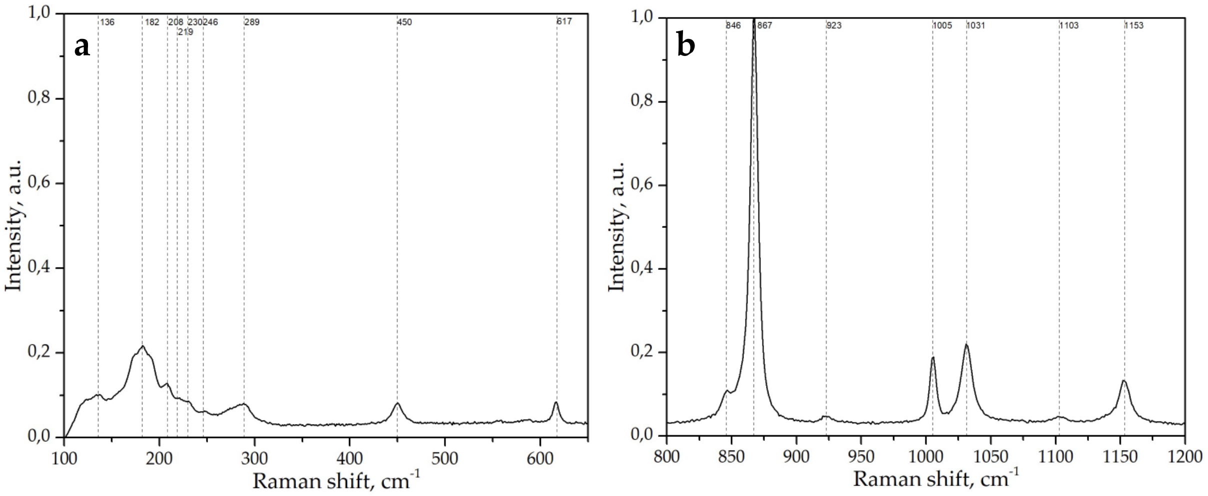

Figure S5 Raman spectrum of $(UO_2)_3(PO_4)_2{\cdot}4H_2O$, recorded at 633 nm. (**a**) 100-650 cm^{-1} region; (**b**) 800-1200 cm^{-1} region.

Table S5 Raman shifts of Cu-phases in the mixture of natural phosphate minerals compared to literature data for Cu-uranyl phosphate and arsenate. Ionic charges are omitted for simplicity.

**Compound**	**Cu-mineral**	**$Cu(UO_2)_2(AsO_4)_2{\cdot}8H_2O$**	**$Cu(UO_2)_2(PO_4)_2{\cdot}8H_2O$**
ν2 (UO_2)	191, 221, 247, 282	218, 240, 276	222, 290
ν2 (AsO_4)	317	320	
ν2 (PO_4)	405, 439,455		399, 406, 439, 464
ν4 (AsO_4)	455	398, 449	
ν1 (UO_2)	820, 855	809, 819	808, 826
ν3 (UO_2)		809, 819	
ν1 (AsO_4)		810 (ν1+ ν3)	
ν3 (AsO_4)	890	888, 910	
ν3 (PO_4)	976, 989, 1000		957, 988, 995, 1004
Reference	This work	Frost&Weier (2004)	Frost&Weier (2004)

Table S6 Raman shifts of Ca-phase in the mixture of natural phosphate minerals compared to literature data for Ca-uranyl phosphates. Ionic charges are omitted for simplicity.

**Compound**	**Ca-mineral**	**$Ca(UO_2)_2(PO_4)_2 \cdot 12H_2O$**	**$Ca(UO_2)_2(PO_4)_2 \cdot 6H_2O$**
ν2 (UO_2)	195, 202	222, 291	222, 263
ν2 (PO_4)		399, 406, 439, 464	387, 453
ν4 (PO_4)		629	507, 643
ν1 (UO_2)	796,818,836	816, 822, 833	818, 833,850
ν3 (UO_2)	860		
ν1 (PO_4)			
ν3 (PO_4)	989, 1013,1027	988, 1007, 1018	989, 1007, 1018, 1033, 1093
Reference	This work	Frost&Weier (2004)	Frost&Weier (2004)

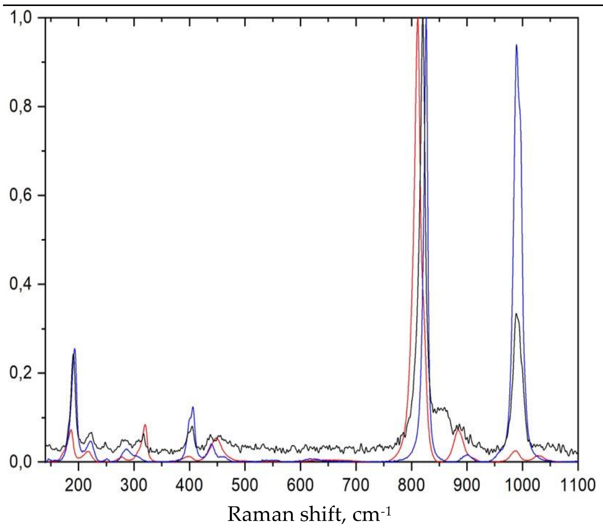

Figure S6 Comparison of Raman shifts of single-phase metazeunerite $Cu(UO_2)_2(AsO_4)_2 \cdot 8H_2O$ *(*RRUF ID: R050524) - red, torbernite $Cu(UO_2)_2(PO_4)_2 \cdot 8H_2O$ (RRUF ID: R070509) – blue, and Cu-mineral of the natural mixture - black.

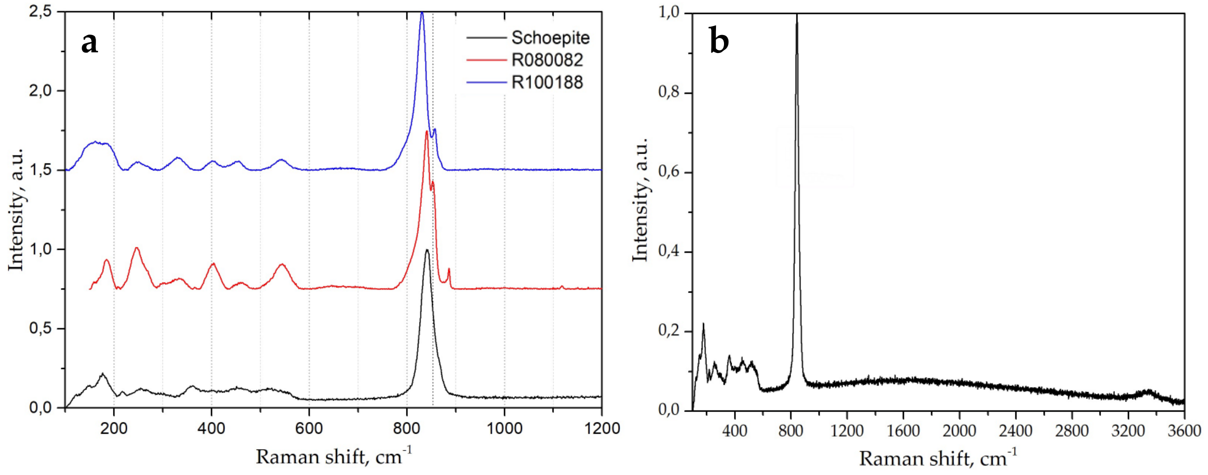

Figure S7 Raman spectrum of metaschoepite, recorded at 633 nm. (**a**) Comparison with data from RRUF Database; (**b**) spectrum in 100-3600 cm^{-1} region.

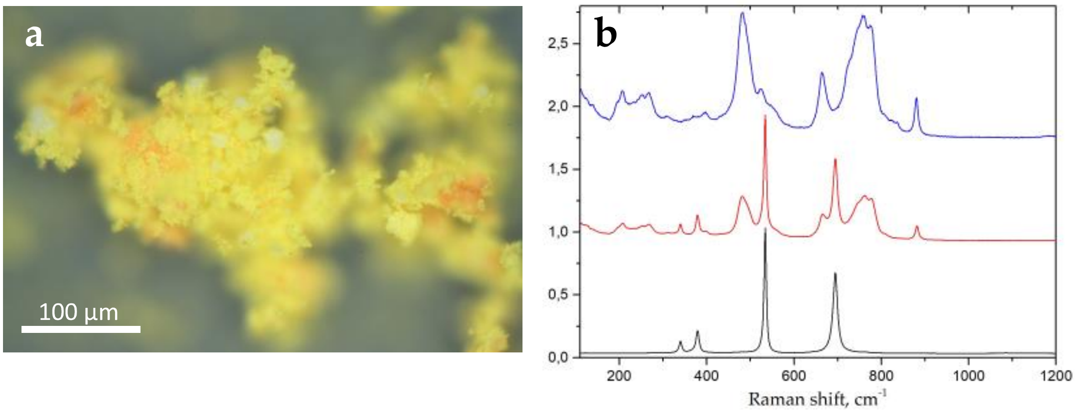

Figure S8 (**a**) Optical image of $CaUO_4$ powder. Yellow crystals are of $CaUO_4$, orange – of CaU_2O_7 impurity; (**b**) Raman spectrum of CaU_2O_7 (blue), CaU_2O_7 impurity in $CaUO_4$ (red), $CaUO_4$ (black), recorded at 633 nm.

Table S7 Raman shifts of Ca uranates compared to literature data for Ca and alkali-metal uranates. Ionic charges are omitted for simplicity.

**Compound**	**$CaUO_4$**	**$CaUO_{4-x}$**	**Li_2UO_4**	**Na_2UO_4**	**K_2UO_4**	**Rb_2UO_4**	**Cs_2UO_4**
			225, 248, 273, 280	238	221	230	238
	340	267	321, 350, 366	329, 362	293, 344		339, 369
	379	351	425, 446, 475, 491	442	439, 492	444	446
	534	533		506, 547			
	695	695	675, 710	712, 736	694	682	713,736
**Compound**	**CaU_2O_7**	**CaU_2O_7**	**$Li_2U_2O_7$**	**$Na_2U_2O_7$**	**$K_2U_2O_7$**	**$Rb_2U_2O_7$**	**$Cs_2U_2O_7$**
	206, 251, 267			202, 233, 274	245, 267, 287	235, 247, 285	224, 258, 270, 292
	308, 369			313, 357	336	331	312, 340, 371
	398, 483		427	420	434, 491	442, 483	425, 474
	525			536	562	547	
	664		635	584, 599			
	725			752			713,736
	758, 780		765, 793	779, 788	778	778	778, 794
	881			826			818
Reference	This work	Allen& Griffiths (1979)	Volkovich et al. (1998)			Volkovich et al. (2001)	

S4. XANES

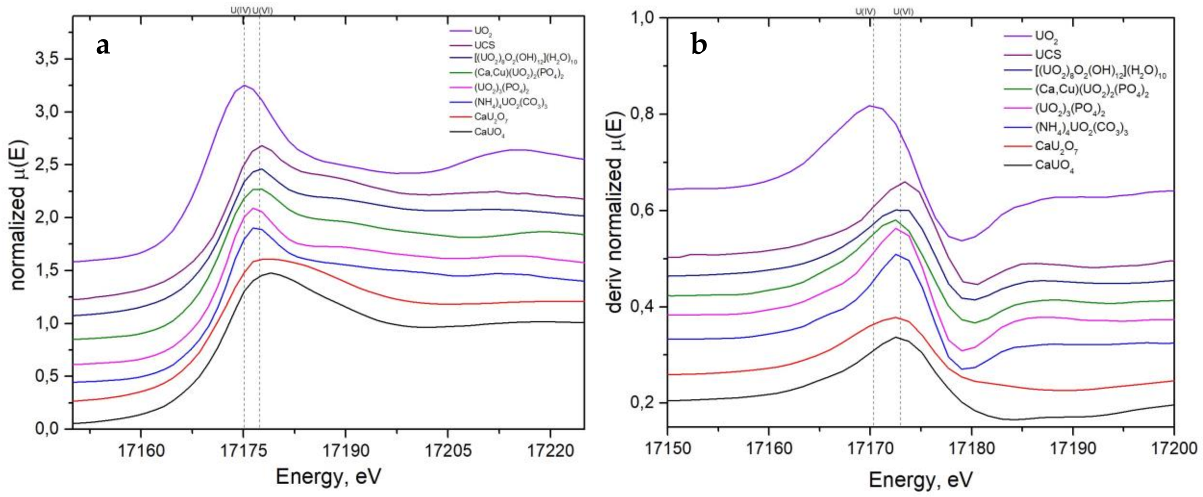

Figure S9 **(a)** U L_{III}-edge XANES spectra; **(b)** normalized derivatives of U L_{III}-edge XANES spectra of studied compounds and references: UO_2 (U^{4+}), metaschoepite (U^{6+}). XANES spectrum of UCS sample was presented and discussed in Maryakhin et al. (2021).

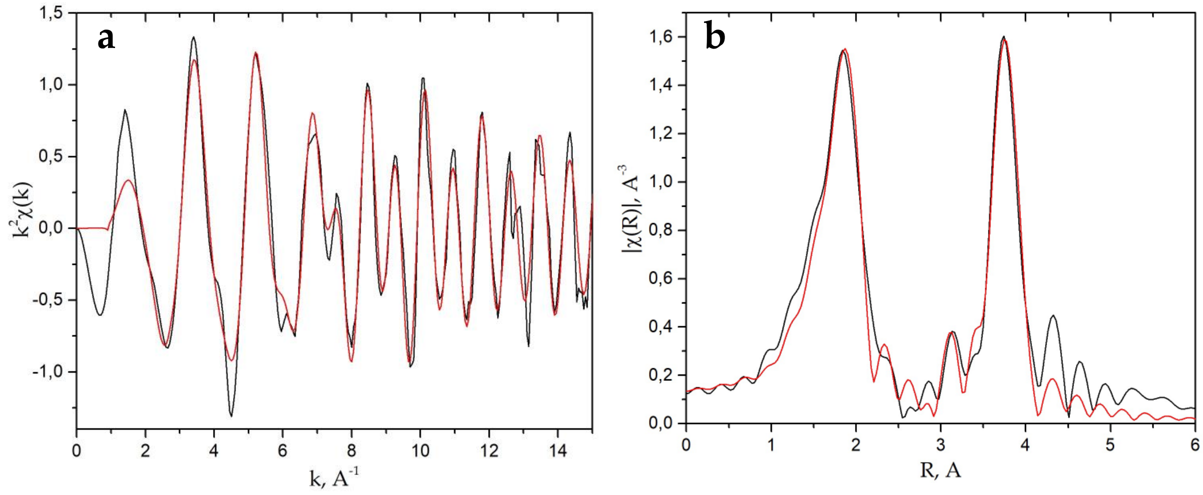

Figure S10 **(a)** U L_{III}-edge edge k^2-weighted EXAFS spectrum of UO_2; **(b)** Fourier transform magnitude (k range 3-15), not corrected for phase shifts. Black lines are experimental data, red lines represent fit curves.

Table S8 Fitting of UO_2 spectrum in comparison to literature data.

U-O (1)			U-U			U-O (2)			Reference
CN	R, Å	σ^2, Å^2	CN	R, Å	σ^2, Å^2	CN	R, Å	σ^2, Å^2	
8	2.37		12	3.86		24	4.53		Wasserstein (1951)
7.8±0.5	2.35±0.01	0.009	11.4±4	3.87±0.01	0.005	22.8±7.9	4.49±0.02	0.01	O'Loughlin et al. (2003)
8.1±2.4	2.34		6.3±1.9	3.86					Opel et al. (2007)
8	2.38	0.003	12	3.90	0.005				Denecke et al. (2005)
8	2.37±0.01	0.007	12	3.88±0.01	0.004	24	4.54±0.04	0.017	Boyanov et al. (2007)
8	2.34±0.01	0.012	6.2±0.8	3.85±0.02	0.007				Boyanov et al. (2017)
8	2.34	0.008	12	3.85	0.005	24	4.49	0.009	Jovani-Abril (2014)
8	2.34	0.004	12	3.87	0.004	24	4.52	0.008	This work

S5. References

Allen, G.C.; Griffiths A.J. (1979). *J.C.S. Dalton*, **2**, 315–319.

Armstrong, C.R. (2009). Publisher: Washington, USA. pp. 33–37.

Boyanov, M.I.; Latta, D.E.; Scherer, M.M.; O'Loughlin, E.J.; Kemner, K.M. (2017). *Chem. Geology*, **464**, 110–117.

Boyanov, M.I.; O’Loughlin, E.J.; Roden, E.E.; Fein, J.B.; Kemner, K.M. (2007). *Geochim. et Cosmochim. Acta*, **71**, 1898–1912.

Denecke, M.A.; Janssens, K.; Proost, K.; Rothe, J.; Noseck, U. (2005). *Environ. Sci. Technol.*, **39**, 2049–2058.

Faulques, E.; Perry, D.L.; Kalashnyk, N. (2018). *Vibrational spectroscopy*, **99**, 184–189.

Frost R.L.; Carmody O.; Erickson, K.L; Weier, M.L.; Cejka, J. (2004). *J. of Molecular Structure*, **703**, 47–54.

Frost, R.L.; Weier, M. (2004). *Spectrochimica Acta Part A*, **60**, 2399–2409.

Jovani-Abril, R. (2014). Doctoral thesis. Universidad de Santiago de Compostela (USC), Spain; Institute for Transuranium Elements (ITU), Germany.

Maryakhin, M.A.; Vlasova, I.E.; Varlakova, G.A.; Germanov, A.V.; Varlakov, A.P.; Kalmykov, S.N.; Petrov, V.G.; Pomanchuk, A.Yu.; Yapaskurt, V.O.; Trigub, A.L. (2021). *Radiochemistry*, **63**, 119–126.

O'Loughlin, E.J.; Kelly, S.D.; Cook, R.E.; Csencsits, R.; Kemner K.M. (2003). *Environ. Sci. Technol.*, **37**, 721–727.

Opel, K.; Weiß, S.; Hübener, S.; Zänker, H.; Bernhard, G. (2007). *Radiochim. Acta*, **95**, 143–149.

Pham-Thi, M.; Colomban, Ph. (1985). *J. of the Less-Common Metals*, **108**, 189–216.

Volkovich, V.A.; Griffiths, T.R.; Fray, D.J.; Fields, M. (1998). *Vibrational Spectroscopy*, **17**, 83–91.

Volkovich, V.A.; Griffiths, T.R.; Thied, R.C. (2001). *Vibrational Spectroscopy*, **25**, 223–230.

Wasserstein, B. (1951). *Nature*, **168**, 380.
